# Machine Learning Algorithms and Fault Detection for Improved Belief Function Based Decision Fusion in Wireless Sensor Networks

**DOI:** 10.3390/s19061334

**Published:** 2019-03-17

**Authors:** Atia Javaid, Nadeem Javaid, Zahid Wadud, Tanzila Saba, Osama E. Sheta, Muhammad Qaiser Saleem, Mohammad Eid Alzahrani

**Affiliations:** 1 Department of Computer Science, COMSATS University Islamabad, Islamabad 44000, Pakistan; atiajavaid477@gmail.com; 2 Department of Computer Systems Engineering, University of Engineering and Technology Peshawar, Peshawar 25000, Pakistan; zahidmufti@nwfpuet.edu.pk; 3 College of Computer and Information Sciences, Prince Sultan University, Riyadh 11586, Saudi Arabia; tsaba@psu.edu.sa; 4 College of Science, Zagazig University, Zagazig 44511, Egypt; uelshata@zu.edu.eg; 5 College of Computer Science and Information Technology, Al Baha University, Al Baha 65525, Saudi Arabia; muhammad.qaiser.saleem@gmail.com (M.Q.S.); meid@bu.edu.sa (M.E.A.)

**Keywords:** Wireless Sensor Networks, machine learning classifiers, KNN, ELM, SVM, RELM, belief function

## Abstract

Decision fusion is used to fuse classification results and improve the classification accuracy in order to reduce the consumption of energy and bandwidth demand for data transmission. The decentralized classification fusion problem was the reason to use the belief function-based decision fusion approach in Wireless Sensor Networks (WSNs). With the consideration of improving the belief function fusion approach, we have proposed four classification techniques, namely Enhanced K-Nearest Neighbor (EKNN), Enhanced Extreme Learning Machine (EELM), Enhanced Support Vector Machine (ESVM), and Enhanced Recurrent Extreme Learning Machine (ERELM). In addition, WSNs are prone to errors and faults because of their different software, hardware failures, and their deployment in diverse fields. Because of these challenges, efficient fault detection methods must be used to detect faults in a WSN in a timely manner. We have induced four types of faults: offset fault, gain fault, stuck-at fault, and out of bounds fault, and used enhanced classification methods to solve the sensor failure issues. Experimental results show that ERELM gave the first best result for the improvement of the belief function fusion approach. The other three proposed techniques ESVM, EELM, and EKNN provided the second, third, and fourth best results, respectively. The proposed enhanced classifiers are used for fault detection and are evaluated using three performance metrics, i.e., Detection Accuracy (DA), True Positive Rate (TPR), and Error Rate (ER). Simulations show that the proposed methods outperform the existing techniques and give better results for the belief function and fault detection in WSNs.

## 1. Introduction

Wireless Sensor Networks (WSNs) collect the data from the physical environment, and the collected data could be of any type. Then, these collected data are further processed by the network. The WSN topology consists of many nodes deployed in many different patterns. It may be random, specific, dense, or sparse deployment. WSN can be applied in many fields due to the rapid development of this field. WSN is playing an important role in other fields, like data science, where there is a huge amount of data to be processed. To classify this huge amount of data from different sensors, authors have used machine learning algorithms. Then, making use of ensemble learning algorithms, prediction results of all the sensors are aggregated. This process of fusing the prediction results of the machine learning algorithms minimizes the problem of selecting the wrong models [1].

Decision fusion has played an important role in WSN for multiclass classification problems. WSN have a number of sensor nodes, which may be static or distributed in the network. These nodes have to communicate with each other directly or indirectly. There is a central node where the data are merged and the decisions are made about the sensor data. If the sensors are decentralized, there is a problem of data accuracy. Local sensors have no such capabilities to deal with data accuracy issues. Therefore, there is a need for a fusion rule to fuse only those data that are reliable. A decision fusion rule has been proposed to analyze the local sensor data detection and the false alarms generated to confirm if the data are accurate or not [2].

The authors in [3] have used the belief function fusion approach and an estimation classifier to produce the forest image classification method. A remote sensing classification problem of images was tackled, and data were collected from the forests. A belief function theory is used to tackle this classification issue. The distance estimation model classifier was used to calculate the distance of the target. In the WSN, sensors are distributed, sensing data are classified, and the results are sent to the fusion center in binary form. To get the accurate local sensor detections, a threshold is set, and the detections are compared with the defined threshold to reach the final decision.

The decision fusion problem in cooperative WSNs is tackled through the optimal decision fusion rule. This optimal fusion rule is derived in the Neyman–Pearson sense. The optimal decision fusion rule uses the decode-and-forward relaying for the channels. To handle this decision problem, many techniques have been proposed in the literature, and the multi-attribute decision fusion model based on Intuitionistic Fuzzy Set IFS is constructed through two algorithms: the data distribution-based IFS construction algorithm and the category similarity weight-based TOPSIS intuitionistic fuzzy decision algorithm [4].

Decision fusion [5] is used to find the needed amount of energy for the WSN, and the working condition of the scenario is based on on-off keying and statistical channel state information. The optimality of multi-input and multi-output decision fusion is based on energy detection over a class of fading distributions modeled through a Gaussian mixture with zero-mean component. The limited power battery supply of the sensor nodes is the major problem in WSNs. To overcome this problem, multiple classifiers and techniques are used to handle energy dissipation-related issues. Deep learning algorithms and the classifiers had played a major role in the field of WSN. Previous research showed that decision trees are the baseline for the classifiers; constructed on the basis of Fourier spectra, these classifiers are more competent than the deep tree-based structures. Fourier-based classifiers outperform the stream base classifiers [6]. Data loss, aggregation error, and calibration fault are some of the reasons for faults in WSNs. Machine learning is widely used to detect faults in WSNs. There are different types of faults that the research community has faced [7], and they can be categorized according to the sensed data, which are described as follows:

- Offset fault: This type of fault occurs when the sensing unity has done some bad calibrations, which is the reason for the extra constant added to the expected data.
- Gain fault: This type of fault occurs when the change rate of sensed data varies from the expected data in a specific period of time.
- Stuck-at fault: This type of fault occurs when there is no variation in the sensed data, and the sensed data series is zero.
- Out of bounds: This type of fault occurs when there are normal readings and some readings are out of bounds from the normal trend.

Different classification algorithms have several advantages; therefore, it is not easy to decide which classifier is best for decision fusion approaches. Therefore, the belief function-based decision fusion approach is used as it is compatible with any type of classifier. Machine learning algorithms are used to improve the fusion accuracy of the belief function fusion approach. In this study, we aim to use the methods that will reduce the complexity of the combination operation and the energy consumption of the nodes [8]. In the previous approaches, the complex Basic Belief Assignment (BBA) combination operation required each sensor node to upload the whole BBA structure to the fusion center, which consumes a high amount of energy in data transmission. The belief function fusion approach is used, which adds less burden on the nodes and the fusion center. In the fusion center, Dempster’s combinational rule is used to acquire the unified BBA, which is a simple combinational rule. WSNs are prone to errors and failures, so the SVM classifier is used to detect failures [7]. We have used Enhanced Support Vector Machine (ESVM), Enhanced K-Nearest Neighbor (EKNN), Enhanced Extreme Learning Machine (EELM), and Enhanced Recurrent Extreme Learning Machine (ERELM) for fault detection to get better fault detection results.

### 1.1. Motivation and Problem Statement

The decentralized belief function-based decision fusion approach is used to improve classification results by uploading as little data to the fusion center as possible. A BBA construction algorithm, based on the training output confusion matrix, and BBA combination under Dempster’s combinational rule of the belief function theory are used for our scenario. Classifiers are proposed, to improve the belief function-based decision fusion approach. In WSNs, decentralized target and multi-class decision fusion are the main causes of facing problems in aggregating decisions of several different classification systems. For this problem, the belief function-based decision fusion approach is used [8]. Target classification is the main problem in WSNs [2]. Classifiers are enhanced to improve the classification results, so that fusion results could be enhanced. This helps to achieve an improved belief function-based decision fusion approach. On the other hand, WSNs are prone to errors and failures, so the SVM classifier is used to detect failures. Fault detection, using four different faults [7], is done to check the detection accuracies of the proposed classifiers. We have used ESVM, EKNN, EELM, and ERELM for fault detection to get better fault detection results. In WSNs, during data transmission, if any node becomes faulty, the fusion center will not receive the needed data. In order to analyze if nodes are faulty or sending faulty data, it is necessary to perform fault detection. Four types of faults are induced to analyze if our system is fault tolerant or not using machine learning algorithms.

### 1.2. Contributions

The contributions of this paper can be described as follows:

1. EELM, EKNN, ESVM, and ERELM are proposed for the classification purpose.
2. We have improved the existing belief function-based decision fusion approach.
3. To analyze the DA of enhanced classifiers, we have induced four different faults: offset fault, gain fault, stuck-at fault, and out of bounds fault) in our dataset and then used four different classifiers (EKNN, EELM, ESVM, and ERELM) to detect faults in our scenario.
4. Then, we have calculated the ER for the enhanced classifiers.
5. Then, we calculated the TPR for each enhanced classifier to analyze how classifiers are performing in our scenario.
6. We have tested the classifiers on two datasets, the sensit vehicle dataset and on the other dataset that was prepared by inducing four different faults.

The paper is organized as follows: Section 2 consists of the related work. Section 3 provides a discussion about the system model. Section 3.1 provides a discussion about belief function theory. Section 3.2 provides a discussion about fusion rules. Section 4 provides a discussion about existing and enhanced classifiers. In Section 5, faults and performance metrics are discussed. In Section 6, simulation results are given for both experiments. Finally, the paper is concluded in Section 7.

## 2. Related Work

Many research contributions have been made in the field of machine learning algorithms in WSNs. Authors have explored the performance of Convolutional Neural Network (CNN) classifiers for the golf swing data classification method. The classifier is tested on the basis of accuracy. The 10-fold cross-validation method is used to test the performance of CNN classifier, which indicates that the CNN classifier best fits the classification of golf swing data classification. CNN is compared with the SVM classifier where SVM achieves 86.8% accuracy and CNN 95.0% [9]. WSN have the power of interfacing with other fields like home automation, environment monitoring, ship hull monitoring, underwater acoustics, and biomedical diagnosis. Artificial intelligence concepts are the base of machine learning techniques that are utilized in computational tasks where conventional techniques cannot be applied. In [10], the author has used many machine learning algorithms on different ranges of datasets and tested the classification accuracy of Naive Bayes (NB), linear regression, and Stochastic Gradient Decent (SGD). Classifiers were used for the classification purpose, and Gaussian naive Bayes outperformed the other classifiers. ECG signal preprocessing and SVM were combined to classify the data. To reduce latency and computational overhead, the adaptive filter was used. Pricipal Component Analysis (PCA), Artificial Neural Network (ANN), a knowledge-based system, KNN, and SVM were used with parameters such as ECG and HRV for classification in [11].

The heterogeneous ensemble approach and objective function were used for the proposed Advanced Extreme Learning Machine (AELM) technique. The proposed AELM is based on the regularized-ELM, $L_{2}$-norm-optimized ELM $\left(ELML_{2}\right)$, and kernel-ELM in [12]. Using the WMV, class labels were identified to define different decision boundaries. When ER was reduced using the proposed approach, the classification accuracies and the fusion accuracies were increased. In this paper, the authors have proposed a classification protocol for the decision trees, and the main contribution was the enhancement of the hyperplane-based classifiers by combining the building blocks of the proposed approach. The accuracy of the enhanced approach was tested on the UCI repository datasets.

In [13], the authors have implemented Multilayer Perceptron (MLP) to predict the wireless sensor outputs. Structured MLP was presented, in which the first layer was comprised of only one node, which received the inputs from all components. The outputs and inputs of all the layers were connected. MLP contains special learning algorithms that will produce the values for the weights to handle different situations. Using different layers of MLP, different advantages can be gained. The paper discussed how the two models were constructed using MLP. The scheme was based on developing a monitoring framework for streaming data analysis in the field of deep learning for WSN. There are many reasons why sensor data are not accurate: it might be incomplete or have some outliers. Because of these reasons, if one wants to work on accurate data to get accurate results, deep learning algorithms are needed. The proposed method in [14] makes queries to get the accurate results along with the maintained accuracy even if there is some missing data because it satisfies the time constraints. Real-world data were used to confirm the accuracy of deep learning algorithms. Pan et al. proposed the KNN based missing data estimation algorithm, which is an enhanced version of KNN, to predict the data accurately.

The true status of the primary user was taken from the environment for the machine learning-based reliable spectrum sensing technique. Sensors sensed the data and stored these data into sensing classes. Sensing classes were made for these data, and recent sensing data were stored into one of these classes. From the classification results, it was predicted that primary user was present or absent. The decision combination scheme combined the local decisions at the fusion center on the basis of the reliability of the cognitive radio users in [15]. There are many problems in the field of WSN that are handled through deep learning and classification algorithms. In [16] the authors have defined an SVM algorithm to deal with such a problem. Data sensors when sensing inaccurate data passed this inaccurate and erroneous data to the application, which can result in huge damage. Therefore, in order to cope with such issues, the SVM classifier was used, which separates the accurate and faulty data. Because of the kernel functions used, this method has the adaptation power to deal with nonlinear classification.

In [17], the authors were dealing with four faults, namely: erratic, drift, hard-over, spike, and stuck faults. The dataset was induced with the faults, and for each fault, 100 samples were prepared that contained 1000 data elements in each dataset. SVM was used for classification with three different kernel functions, namely: linear, polynomial, and radial-basis function kernels. The paper discussed that increasing the number of training data increased the efficiency of the classifier; however, the problem of over-fitting was raised. To tackle the problem of over-fitting, K-fold cross-validation was adopted. In this study, the authors worked on noise fault, short-term fault, and fixed fault, which were caused by the low battery and bad calibrations. To identify and classify the faults, the authors have used SVM, NB, and gradient lifting decision tree and made a comparison among them. Decision fusion with distributed co-operation and large number of sensors is discussed in [18,19]. Deep learning and belief function techniques are used for classification of forest images, surgical site infections and real time data [3,20,21]. Authors have done fault detection using different techniques like particle swarm optimization, SVM, NB, and neural networks [22,23,24]. Gray wolf optimization technique is used to enhance recurrent extreme learning machine for the classification purpose [25]. Authors have used different techniques to analyze the behaviour of large number of sensors in WSNs [26,27,28,29].

In [30], a survey was conducted for the machine learning techniques considering the application and networking perspective in WSNs. Machine learning techniques are used to solve many problems like energy-aware communication, sensor deployment, resource allocation, and task scheduling. Machine learning techniques are especially used for information processing, such as data conditioning and machine inference in the application domain. Limitations and future directions of the learning algorithms were discussed. In [31], a survey of machine learning techniques for routing problems in WSNs is presented. This survey provided guidelines to the researchers who want to apply machine learning techniques in the field of WSNs. This survey also provided existing and on-going research of machine learning techniques in WSNs. In [32], the KNN query processing algorithm was used to compute an energy-efficient sensor boundary that enclosed at least *k* nearest nodes. The authors have proposed a cost-efficient solution for 3D sensor network. Mobile data collectors were used to compute KNN queries. A 3D plane rotation algorithm was used to map selected sensor nodes on different planes. Then, the authors proposed a neighbor selection algorithm that uses KNN metric to select the KNNs.

Distributed WSNs sense the environmental changes in co-operation with each other. Storage, computational constraints, and limited power supply are some of the challenges faced by WSNs. Computational Intelligence (CI) has been used in recent years to cope with these challenges. CI is flexible and robust to communication failures and topological changes. A survey about the CI application in the field of WSNs was done in [33]. The authors in [34] have addressed the problem of distributed estimation in WSNs. In the case of link or node failures, the distributed estimation approach has shown good performance. The authors have proposed diffusion-based adaptive solutions of the LMS type. In [35], the authors have reduced the communication overhead in WSNs. A backoff-based broadcast scenario was introduced to repeat broadcasts and to handle communication overhead. The localization performance of nodes was also improved using the particle filtering method. Table 1 shows the summary of the related work.

In this paper, we aim to increase the performance of the decision fusion approach by applying four machine learning classifiers, i.e., KNN, ELM, SVM, and RELM, and their enhanced versions, i.e., EKNN, EELM, ESVM, and ERELM. Their simulation results show that the proposed enhanced versions of the classifiers beat the results of KNN, ELM, SVM, and RELM. Then, these classifiers are further used in Experiment 2 to analyze their detection accuracies. For this test, the original dataset was induced with four types of faults: offset fault, gain fault, stuck-at fault, and out of bounds fault. Simulation results show that ERELM achieved the top rank in DA.

## 3. System Model

**Figure 1:**
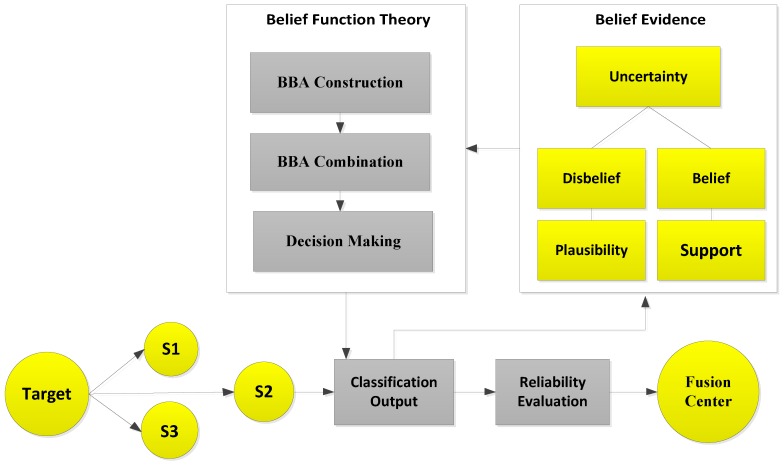
System model of belief function theory.

The sensors were distributed in the network as shown in Figure 1. The sensor senses the target (vehicle), which is shown in the model as our taken observations. Sensors then perform classification for the target classes, and the output of the classification is called the local decision (u) for each observation. Then, this classification result is processed according to belief function theory. In belief function theory, the general belief and value of evidence about every observation are measured. Regarding the uncertainty of the target class, the plausibility (probability) and support functions are calculated according to the belief and disbelief about the observation. Then, these probabilities and general beliefs are forwarded to the belief function theory, which involves three main steps. BBA is constructed on the basis of distance intervals between the target and each sample of the class.

After the BBA’s are constructed, they are combined through the combinational rule of belief function theory. For applying belief function theory, BBAs must be reasonable. After BBA’s construction, probability vectors are needed. Probability vectors of sensors are obtained from the confusion matrix. The decision $u_{i}$ we have is the probability vectors in which $p_{i}\left(u_{i}|w_{k}\right)$ is the conditional probability of the class label $w_{k}$. The larger the probability, the larger the belief will be. These decisions are called hard decisions, and then, the reliabilities of these hard decisions are calculated according to the assumption. We have assumed that if the distance of the target from the sample set of the class is greater, then the reliability, i.e., the target belongs to that specific class, is less if the distance is smaller than the target belonging to that class; for example, if we use KNN classifier and want to know the reliability of the local decision. Then, the distance between the object and KNN is used to know the reliability in the sample set of each class label. If the relative distance $d_{i,j}$ is large, we can conclude that $w_{j}$ is not the class label of the target class. On the other hand, if $d_{i,j}$ is small, there is more possibility that $w_{j}$ is the class label of the target. Then, the local decisions and the reliability measures are sent to the fusion center for global decision making. Then, in the fusion center, the classification results are merged, and final decision is made according to the fusion rule, i.e., belief function theory. The final decision is made on the basis of the class having the maximum BBA’s. Then, we have used the enhanced classifiers: EKNN, EELM, ESVM, and ERELM, to enhance the belief function theory, which outperformed the existing belief function theory, NB, and WMV because of its reliability evaluation of the local decisions.

In the second part of the system model, we induced four types of faults in our dataset to analyze two things: how our enhanced classifiers performed in the case of faulty data and how belief function theory performed in the case of faulty data. Sensor data faults directly affect the final decisions made by the fusion center. In order to avoid this condition, we have done fault detection for this study. For fault detection, the sensed readings are sent to the data preparation block. The next step is the induction of faults in the datasets. Datasets with four faults: offset fault, gain fault, stuck-at fault, and out of bounds fault, were prepared. The techniques we have used for fault detection were EKNN, EELM, ESVM, and ERELM. These techniques use observation vectors to formulate a decision function. WSN is composed of multiple clusters of nodes interconnected with each other. For the learning phase, labeled datasets were used, and they were composed of a set of normal and faulty data. After deployment of the decision function in the cluster head, data were classified into two classes. If the result was positive, the data belonged to the first class (normal case); otherwise, data belonged to abnormal class (faulty case). The main contribution of the paper is the enhanced belief function fusion approach using the proposed enhanced classifiers. To enhance the existing belief function fusion approach, classifiers were enhanced, and through these enhanced classifiers, classification and fault detection were done. To analyze to what extent the existing belief function fusion approach was improved, we included fault detection in our scenario of decision fusion. If the fusion approach is used, the first priority is to send accurate results to the fusion center. To send accurate local decisions to the fusion center, the necessary steps were taken: classification, fault detection, and reliability of the local decision. Classification and fault detection were done using the proposed enhanced classifiers. This saves the time and energy of the fusion center in checking whether the results were accurate or not and reduced the risk of fusing inaccurate results, because we were sending a three-step checked local decision to the fusion center.

### 3.1. Belief Function Theory

Belief function theory is also named Dempster–Shafer evidence theory, which provides a solution for the multisource information and decentralized target. This approach fuses the results from different sources to make a final decision. It reduces the burden of considering all the data and also deals with uncertainty. This approach uses the classifiers’ training output confusion matrix for construction of BBAs for each sensor. Real-time observations are also used in the construction of BBAs for sensors. The reason for using this fusion approach is that other fusion techniques rely on the sensors to upload the whole BBA structure to the fusion center. While in belief function theory, the whole BBA structure is not uploaded to the fusion center in order to reduce the burden of the fusion center and to reduce the energy consumption in transferring all the data. A global BBA was prepared under the Dempster’s combinational rule in the fusion center. Belief function theory includes two phases: mass construction and BBA combination [8].

#### 3.1.1. BBA Construction

For mass construction, we have used the frame of judgment (having possible answers), which includes the finite non-empty set, which is mutually exclusive. We assumed that Ω = $A_{1}$, …, $A_{c}$ is our frame of judgment, and its power set is $2^{\Omega }$. The mass function of $2^{\Omega }$ is $2^{\Omega }\to \left[0,1\right]$, which satisfies the following equation [8].

(1) $$\sum_{A\subset 2^{\Omega }}m\left(A\right)=1\;\;m\left(\phi \right)=0$$

where *A* is a subset of $2^{\Omega }$ and m(A) is basic belief assignment that shows the rank of the subset A. There are two metrics that define the reliability of the hypothesis A, which are given below [8].

(2) $$Bel\left(A\right)=\sum_{A_{i}\subset A}\;m\left(A_{i}\right)$$

(3) $$Pls\left(A\right)=\sum_{A_{i}⋂A\neq \phi }m\left(A_{i}\right)$$

Bel(A) indicates belief. For the hypothesis A of the evidence, the support degree is Bel(A); while plausibility Pls(A) is the degree, which is not in conflict with the hypothesis A for the evidence.

#### 3.1.2. BBA Combination

We have used the combinational rule of belief function theory for decision fusion in order to combine the several BBAs into the final BBA. We have calculated the BBAs for the hypothesis A using the following Equation [8].

(4) $$m\left(A\right)=\frac{\sum_{{⋂}_{j=1}^{M}}A_{j}=A\prod_{i=1}^{M}m\left(A_{i}\right)}{1-k}$$

Here, *k* is called the conflict degree of the final BBAs. If the conflict degree is equal to one, it shows that there is high conflict among the combined BBAs. If the case is that the conflict degree is high, then we can conclude that the fusion results are unreliable. Class labels that obtain the highest probability are used to make the final decision by using the final Equation [8].

(5) $$BetP\left(A\right)=\sum_{A_{i}\subset A}\frac{m(A)}{A_{i}}$$

### 3.2. Fusion Rules

The fusion rules are discussed as follows:

#### 3.2.1. NB

The NB fusion method assumes that all decisions are mutually independent. In binary fusion systems, this fusion method is regarded as the optimal fusion rule. In a fusion system with M sensors, $P_{i,k}$ is the probability of label k corresponding to decision $u_{i}$, and the fusion decision is made by choosing the label with maximum fusion statistic, as given by [8].

(6) $$l_{d}=argmax_{l\leq k\leq c}\left\{\prod_{i=1}^{n}P_{i,k}\right\}$$

#### 3.2.2. WMV

WMV denote $u_{i,k}$ (1≤i≤n,1≤k≤c) as the decision on label $w_{k}$ of sensor $s_{i}$. When the target belongs to $w_{k}$, $u_{i,k}$ = 1 and $u_{i,j}$ = 0 (1≤j≤c,j≠k). In weighted majority voting, decision $u_{i,k}$ is weighted by an adjusting coefficient $b_{i}$, and the decision is made by [8].

(7) $$l_{d}=argmax_{l\leq k\leq c}\left\{\sum_{i=1}^{n}b_{i}u_{i,k}\right\}$$

Weight $b_{i}$ can be calculated as:

(8) $$b_{i}\propto log\left(\frac{P_{i}}{1-P_{i}}\right)$$

where $P_{i}$ is the classification accuracy of sensor $s_{i}$. Apparently, a sensor with higher accuracy will be assigned a larger weight. This rule always performs better than the simple majority voting rule.

## 4. Existing and Enhanced Classifiers

Four classifiers, namely, KNN, ELM, SVM, and RELM, are enhanced for classification of data and the detection of faults.

### 4.1. KNN

The KNN algorithm is concerned with the searching of the nearest neighbor of each point of reference [14]. It might use the Euclidean, Manhattan, Chebyshev norm, or Mahalanobis distance function to calculate the distance. The Brute Force algorithm (BF) is also used to search for the nearest neighbor. In classification through the KNN classifier, we have used three nearest neighbors and the Euclidean distance function. In the classification phase, KNN searches the training example and calculates the distance between every sample set of the class label and observations. After that, it recognizes the nearest neighbors and then predicts the final classification output. The weight of a distance function is calculated using the labeled representatives of the unknown instances. Using this distance function, the KNNs of the unknown instances are found. These are actually the labeled representatives of the unknown instances of that distance function. Then, KNN for each of the labeled representatives finds the nearest neighbor utilizing the same distance function. After this, KNN checks which of the instances have the same class as their representatives. After confirmation of the same classes, KNN counts the same classes. All the numbers of representatives are added to get the weight of the distance function. When the weights of all the distance functions are calculated, KNN of the unknown instances are weighted according to the distance of that distance function. The KNN set has the class label of each instance, which is weighted using the weight of the set, to which that instance belongs. The final class of the unknown instance is decided using the EKNN. The final class of the unknown instance is that having the highest weight in all sets of the KNN.

### 4.2. EKNN

To enhance the KNN classifier, we made clusters of the nearest neighbor points. The idea behind this method is to assign data to the most nearest cluster containing the neighbor points. Clustering is used to unveil the natural neighbors of the feature data space. For this purpose, we have divided our dataset into three sets: training, evaluation, and testing set. The functioning of clustering for finding the best cluster centers is as follows:

1. Firstly, clusters of training samples using the clustering ensemble method are made. In the process of the ensemble method, the k-means algorithm is applied, which uses the random initialization to cluster the samples. Using simple majority voting, the class label of the cluster center is identified and combined. For the k-means algorithm, we have used the Euclidean distance function.
2. Then, a new test sample is allocated for the nearest cluster label. Using the evaluation set, the quality of the obtained clusters is analyzed. The label is assigned to the sample using the determined nearest cluster.
3. Clustering and evaluation steps are repeated several times. The process is repeated many times until satisfactory good cluster centers are obtained.
4. Finally, using the simple majority voting, we attained the group of the best cluster centers obtained from the clustering technique as our final classifier.

Accuracy is achieved by making comparison of the ground truth label of the data. Several iterations are done, and the cluster center obtained from any iteration is used for the solution. After the training of the classifier is finished, we validated this classifier on our selected dataset. In this way, the accuracy of the classifier is obtained by utilizing the class labels of the evaluation set. *K* = 5 and 50 clustering ensemble members were used. Euclidean distance function was used for the EKNN classifier using Algorithm 1. *M* is the parameter for the cluster ensemble and shows the size of the classifier ensemble. For parameters with different ensemble sizes, *M* was taken as three and five and also compared with the classification performance of KNN. EKNN with the clustering method had correctly classified the testing samples and obtained a low Root Mean Squared Error (RMSE) of 3.02. K-fold cross-validation was used to validate the performance of the EKNN model using the subsets of available input.

**Algorithm 1** Pseudocode of EKNN1: Input: Original dataset2: Output: Classification and DA3: Load the training and test data4: **for**
*i* = 1 to max iteration **do**5:    Partitioning the training set into *k* clusters6:    Determining the class label of cluster centers using majority voting7:    Evaluating the quality of cluster centers8: **end for**9: Designing the final classifier10: end

#### ELM

ELM is a classification algorithm and the extension of the Single-Hidden Layer Feed-Forward Network (SLFN) [12]. ELM uses non-differentiable activation functions and directly reaches the solution. However, other traditional classic gradient-based learning algorithms use differentiable activation functions and may face problems like the local optimum, an improper learning rate, over-fitting, etc. ELM computes feasible results when a single hidden layer is used; otherwise, there is a problem of over-fitting. In ELM, we used 20 hidden neurons, and the activation function was “radbas”. ELM requires more computational power and shows less efficiency when dealing with huge amounts of datasets. This is the reason for enhancing the ELM classifier.

### 4.3. EELM

In EELM, we have used the sigmoid function, and data were scaled between (−1, 1). Thirty hidden neurons were used in EELM. It combines the top hidden nodes in the previous layer with the newly-generated nodes as the current hidden layer. Here, the bias value was added to check the training accuracy of the classifier. Input weights and biases for EELM were determined using our proposed algorithm. *L* is the number of hidden neurons, and *n* is the training samples. The optimization method used to optimize the parameters of ELM is described as follows:

1. Firstly, we divided the dataset into five subsets. In every subset, there were four training sets and one testing set.
2. We preferred to encode the solution with dimensions of (L×(n+1)). The input weight values were first (L×n) dimensions. Bias values were the other *L* dimensions.
3. Then, we initialized the parameters with random values including hidden layers and hidden neurons.
4. From Step 2, we obtained the weights and biases. We trained the ELM classifier using these weights and biases. We had a vector solution. Further, we had to convert it into a two-dimensional matrix for the training process.
5. Then, we calculated the fitness value using Equation (9).
6. After the calculation of the fitness value, we increased the number of iterations from 200 to 250 and updated the features in every set.
7. After increasing the number of iterations, we trained the ELM classifier again with the weights and biases obtained from Step 6. Then, we calculated the fitness value one more time using Equation (9).
8. A greedy selection process was used to enhance the fitness value. Then, we calculated the probability value for every solution. A solution with the highest fitness value will be selected.
9. Weights and biases obtained from Step 8 were used to train ELM and then again calculate the fitness value for each solution.
10. Repeat Step 8 to enhance the current fitness value.
11. If the fitness value is not enhanced, then increase the number of iterations and calculate the fitness value again for the new randomly-generated solution.
12. Optimal fitness values from previous solutions were compared with the current value of fitness. If current fitness value dominated, the previous value kept that value and solution.
13. If the stopping criteria have been accomplished, then stop; otherwise, move to Step 5.
14. After this process, we obtained the optimal weight and bias in the range of [−1, 1] using Algorithm 2.

The fitness value was calculated using equation below.

(9) $$f=1-\frac{\sum_{i=1}^{n}misclassificationrate_{i}}{n}$$

Then, we calculated the output weights using the Moore–Penrose generalized inverse method using Equation (16). Using K-fold cross-validation for the EELM, we calculated the RMSE value. EELM had a secured RMSE value of 2.49.

**Algorithm 2** Pseudocode of EELM1: Input: Original dataset2: Output: Classification and DA3: begin:4: Initialize: event target is detected by *n* sensors5: **for** Each observation **do**6:    $x_{i}$ (1 ≤ *i* ≤ *n*) is received by sensor $S_{i}$7: **end for**8: Classify the object using EELM9: Randomly generate input weights $w_{i}$ and hidden biases $b_{i}$10: Calculate the fitness value till the solution is achieved11: Calculate hidden layer output matrix H12: Compute the output weights matrix using Equation (16)

#### SVM

For classification and regression problems, SVM is used [11]. A supervised machine learning algorithm uses the kernel technique to convert the data and then uses these conversions to reach one final optimal boundary for the possible outputs. The kernel function maps the inputs to the high-dimensional feature space. SVM separates the data on the basis of labels and outputs an optimal hyperplane, which categorizes the data. SVM is used to analyze the accuracy trend of the belief function approach. Preprocessing is necessary before applying the SVM classifier on the unstructured data. We have used SVM for classification of the sensit vehicle dataset. For SVM, we have used some other features from the dataset and normalized the dataset between [−1,1]. The importance of each feature was analyzed, and then, weights were assigned to them. These weights were added to a kernel function. Then, these weights were used to measure the importance of each feature. Our dataset was unstructured; therefore, we have used sigmoid kernel function, and the weights of the training set were re-weighted. The basic principles of weight calculation were: (1) if a feature was not present, then the weight of this feature was zero; (2) the more times a feature appeared, more important that feature was.

### 4.4. ESVM

The efficiency of SVM entirely depends on the selection of the *C* and *g* parameters of the kernel function. If these parameters are not carefully selected, the efficiency of the SVM will be greatly hindered. To select optimal SVM parameters and to tune these parameters, we have used Genetic Algorithm (GA), which includes the following steps:

1. Initialization: Firstly, for the solution space, we have defined the data error penalty coefficient *C*. Then, parameter *g* was defined, which is the the genotype data arranged in different combinations. Initially, N genotype data were produced and grouped, which were used by the GA as a starting point.
2. Fitness calculation: The feature fitness was calculated to analyze whether each optimization criteria was satisfied or not. After calculating the satisfied optimized criterion and their respective solutions, then the output would be produced. If optimization criteria were not satisfied, then move towards Step 3.
3. Selection: Fitness values were analyzed and the features that had the highest fitness values were moved to the next group. Features with the highest fitness values were selected.
4. Crossover: Features in the group were matched, and their pairs were exchanged depending on the crossover probability conditions.
5. Variation: In this step, variation probability was applied to all features to check which combination of features would provide the solution.
6. Calculation analysis: In this step, we determined whether features in the newly-generated group satisfied the end condition or not. If the end condition was not satisfied, move back to Step 3. Halt the process when the end condition is fulfilled.
7. Now, our SVM model was well trained, and output was the accurate classification result using Algorithm 3.

To optimize the *C* parameter, the range was (1–20). The gamma parameter (*g*) was set as 0.11. The population size was (1–100). The number of validations were (2–5), and the radial basis kernel function was used. For validation purpose, we have used the K-fold cross-validation test. ESVM showed good performance in the K-fold cross-validation test with an RMSE of 2.28.

**Algorithm 3** Pseudocode of ESVM1: Input: Original dataset2: Output: Classification and DA3: Data preprocessing4: Input Parameter: Initialize parameter5: A hypothesis space in the high-dimensional feature space kernel function is used6: Training model7: Fitness calculation8: Satisfactory termination condition9: Optimal parameter (*C*, *g*)10: Model establishment11: Performance evaluation12: end

### 4.5. RELM

RELM is a single hidden layer neural network (SHLRN), which is a feedback intra network that uses output (Jordon ANN) or hidden layers (Elman ANN). Context neurons are connected backward from the output to the input. These neurons perform like input neurons, and they hold delayed values from output neurons. Afterwards, the same learning method was used as was used in ELM for updating weights and biases. Weights and biases were decided randomly. Optimum results against weights and biases were utilized in RELM randomly. Feedback output along with delay acted as an input and was added into the hidden layer *H* matrix [32]. The trained dataset was used to calculate the unknown weights of the hidden layer. Hidden layer weights were calculated by the Moore–Penrose generalized inverse.

### 4.6. ERELM

ERELM is used to analyze the performance of the belief function-based fusion approach, which outperformed the result of the RELM method. Optimum network parameter such as the approximation function in network, the number of neuron, and the context neurons will be finalized. The sigmoid function was used for RELM method. We initialized ERELM with the learned weights of RELM, which were the best representatives of the input data, so it would learn efficiently. The layers of the ERELM are discussed below:

1. Input layer: This is the first layer of the network, and ERELM starts its working from this layer. The input layer does not have the weighted inputs and does not take input from the previous layer. The number of neurons at the input layer is equal to the number of features in the dataset.
2. Hidden layer: This is the second layer of the model. The values from the input layer are passed to the hidden layer. We have used three hidden layers, and the number of hidden neurons on the hidden layers were 10, 20, and 30 for different iterations. The number of hidden neurons were greater than the number of features. The weights and outputs of the previous layer were used for the matrix multiplication to calculate the output of the hidden layer. The bias value was also added to this output.
3. Output layer: The output of hidden layer was the input of this output layer. The sigmoid function was used to convert the output into a probability score of each class.

We have optimized the weights using the differential evolution method using Algorithm 4. The differential evolution method consists of three main steps. First, we initialized our candidate solution, and for every candidate solution at each iteration, we performed the following steps.

1. Mutation: In mutation, we randomly created vectors of three distinct candidate solutions from our dataset.
2. Crossover: Then, we randomly swapped these vectors, and was crossover constant was chosen from range [0, 1]. Swapping will be continued until satisfactory results are obtained and the termination condition is met.
3. Selection: Then, vectors are compared, and best solution is selected.
4. The process is repeated until the goal is achieved.

Output weights were calculated using Moore–Penrose generalized inverse. The fitness function RMSE was used to analyze the fitness value of each vector in the solution. ERELM scored a 0.0887 RMSE value when the hidden neurons were 10. In differential evolution, mutation, crossover, population size, and the number of iterations were specified as 0.8, 0.7, 50, and 3, respectively. The input weights *w* and biases *b* were randomly chosen within the range of [0, 1]. Then, features and targets were assigned to ERELM. The input data were learned in one forward pass, and the weights were not changed during the training process. Therefore, the network would learn efficiently, and if there were an error, ERELM would update the weights and adjust them according to every input. The sigmoid function was found as an optimal transfer function for both RELM and ERELM. Cross-validation was performed using TPR and ER, and these performance metrics showed that ERELM had gained the highest rank in all classifiers.

**Algorithm 4** Pseudocode of ERELM  1: Input: Original dataset  2: Output: Classification and DA  3: Weights are optimized through the differential evaluation method  4: Assign the input weights wi and biases bi as received from RELM.  5: Calculate the hidden layer output matrix *H*, where *H* = *hij* (*i* = 1, …, N) and *j* = (1, …, K) and *hij = g(wj · xi + bj )*  6: Calculate the output weight matrix using the Moore–Penrose generalized inverse of the H matrix through Equation (16)  7: Updated weights will be given as context neurons to the input and hidden layers.8: end

## 5. Faults in WSN and Performance Metrics

**Figure 2:**
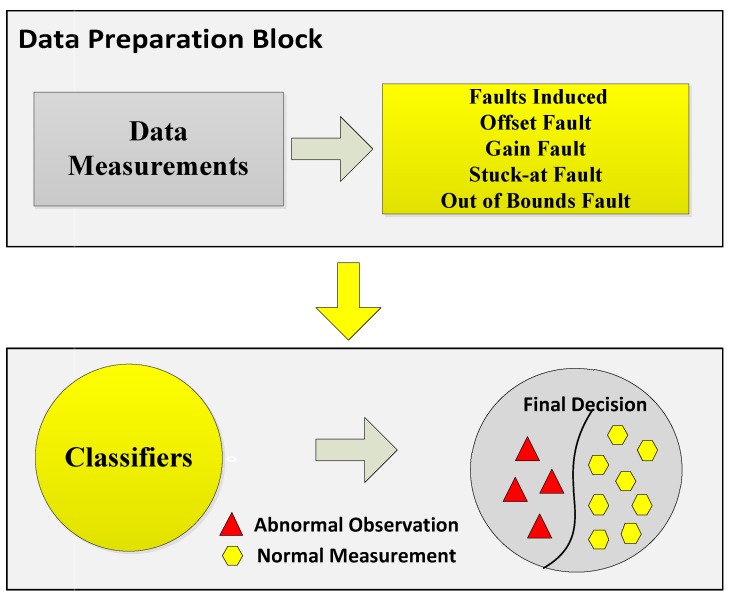
System model of fault detection.

In the second experiment, the sensit vehicle dataset used in the first experiment was induced with four types of faults: offset fault, gain fault, stuck-at fault, and out of bounds fault as shown in Figure 2. These faults were induced in the dataset with different rates of 10%, 20%, 30%, 40%, and 50%. The dataset for fault detection was modeled by using the equation α+βx+η, where α is the offset, β is the gain, *x* is the non-faulty data gathered by the node at time *t*, and η describes the noise in the data [7]. The values for β, α, and η were 0.5, 0.4, and 0.1.

Offset fault was modeled using the equation:

(10) $$x^{\prime }=\alpha +x+\eta$$

Gain fault was modeled using the equation:

(11) $$x^{\prime }=\beta x+\eta$$

Stuck-at fault was modeled using the equation:

(12) $$x^{\prime }=\alpha$$

An out of bounds fault happens when, for sensed data, $x^{\prime }\subset f\left(t\right)$ such that:

(13) $$x^{\prime }<\theta_{1}\;or\;x^{\prime }>\theta_{1}$$

Five datasets were prepared for each fault, and a total 20 datasets were prepared. In the dataset for the normal and faulty observations, the target column shows 1 and −1 classes. The experimental results show that ERELM detected more faults during the data transmission, showing the highest error detected, as shown in Table 2. The DA of ERELM outperformed the DA of other methods. Other methods had less DA because of the distance of the decentralized target, feature selection. To evaluate the performance of EKNN, EELM, ESVM, and ERELM, two metrics were used for comparison. The first metric was DA [7], which can be calculated using the following equation.

(14) $$DA=\frac{\mathrm{number}\;\mathrm{of}\;\mathrm{faulty}\;\mathrm{observations}\;\mathrm{detected}}{\mathrm{Total}\;\mathrm{number}\;\mathrm{of}\;\mathrm{faulty}\;\mathrm{observations}}$$

The second metric used for comparison was TPR [7], which can be calculated using the following equation.

(15) $$TPR=\frac{TP}{TP+FN}$$

**Figure 3:**
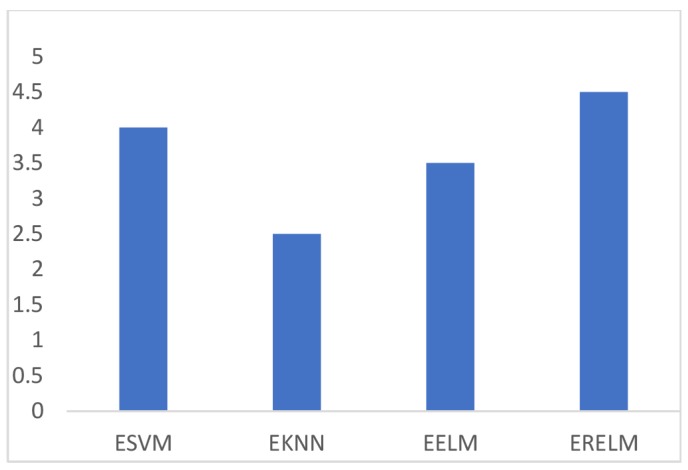
TPR of ERELM, ESVM, EELM, and EKNN.

True Positive (TP) refers to the observations that were correctly identified. False Positive (FP) indicates the observations that were incorrectly identified as negative observations. Figure 3 shows the comparison of TPR among ERELM, EELM, ESVM, and EKNN for four fault data types. ERELM gave a 4.5 TPR, which was higher than other classifiers, which means that ERELM was correctly detecting more actual positives.

**Figure 4:**
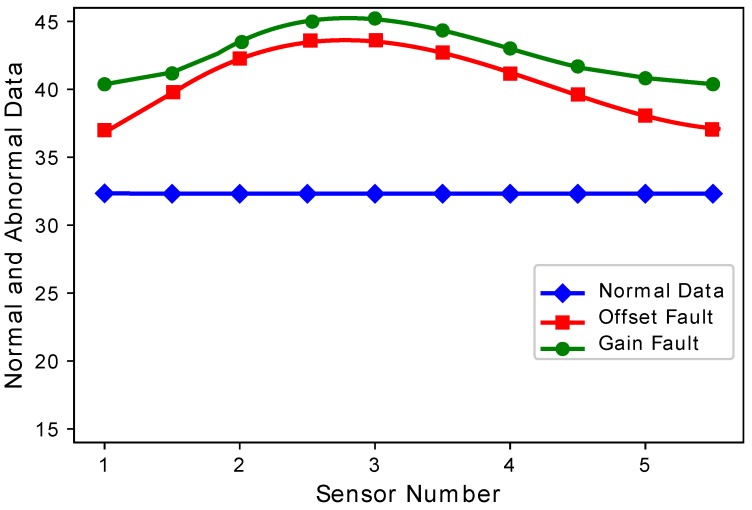
Normal and abnormal sensor data.

**Figure 5:**
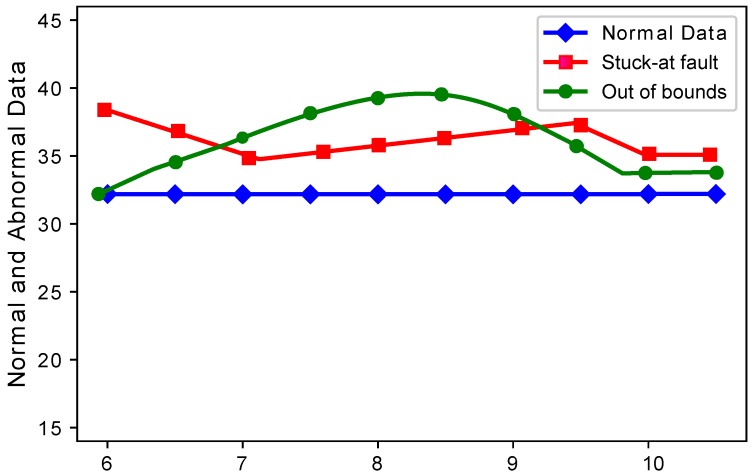
Normal and abnormal sensor data.

Figure 4 and Figure 5 show the difference between normal sensor data and abnormal sensor data. Normal sensor data were compared with abnormal sensor data considering all the faults: gain fault, offset fault, stuck-at fault, and out of bounds fault. Figure 4 shows that every sensor suffered different fault rates. This means that Sensors 2 and 3 sensed more gain faults as compared to Sensor 1, 4, and 5. This happens because there was a constant multiplied with every reading in the gain fault. The offset fault also followed the same trend. Figure 5 shows that when the number of sensors were increased, the stuck-at fault showed a decreasing and increasing trend at Sensors 7, 8, and 9. The stuck-at fault also showed a changing trend because sensors also sensed stuck-at fault at different sensors. The blue lines do not show variations, because sensors sensed normal sensor data. The out of bounds fault also showed an increasing trend because the sensor sensed some values from out of bounds.

## 6. Simulation Setup

**Figure 6:**
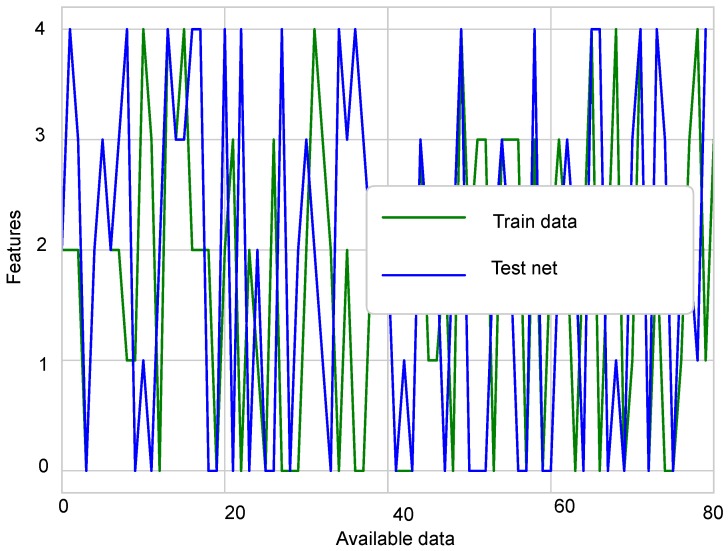
Training data.

**Figure 7:**
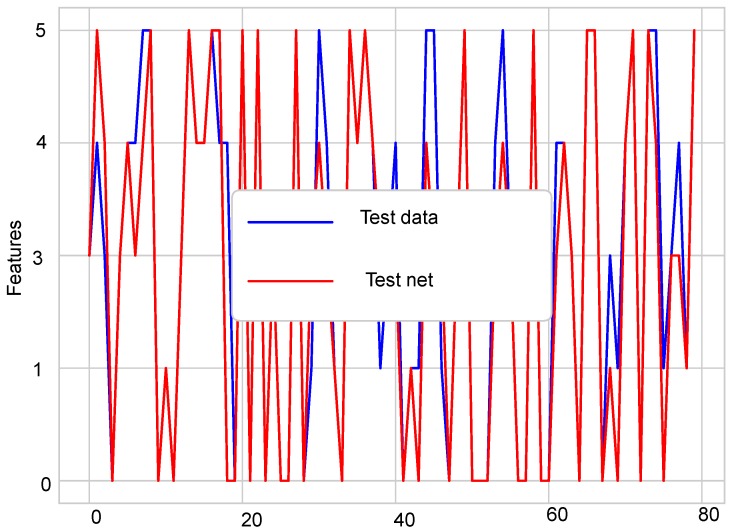
Testing data.

For simulation purposes, MATLAB was used for the first experiment, and for the second experiment, the Python environment was used. Simulations were run on an Intel Core i3, having 4 GB RAM and 500 GB storage. The sensit vehicle classification dataset was used for classification. For vehicle classification, 11 sensor nodes were used in the network. In the experimental section, two experiments were conducted. The first experiment was used to analyze the fusion performance of the belief function fusion approach using the sensit vehicle dataset; for which four different classifiers were used and different comparison analysis were made. In the second experiment, the dataset used in the previous experiment was induced with four type of faults; offset, gain, stuck-at, and out of bounds, to check the detection accuracies of enhanced classifiers. For simulations, the dataset was divided into training and testing data. Eighty percent of the data were used as the training data and 20% as the testing data as shown in Figure 6 and Figure 7. Testing and training graphs are given below.

### Results and Discussion

**Figure 8:**
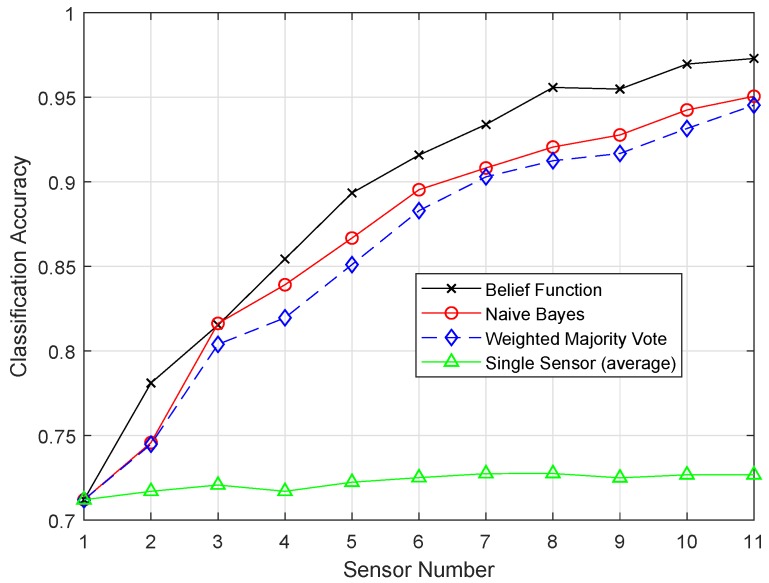
KNN.

**Figure 9:**
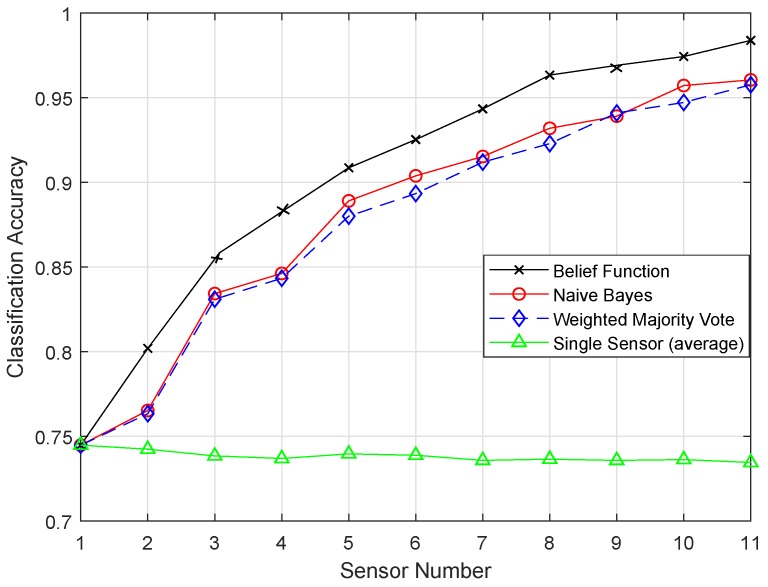
EKNN.

The original versions of the classifiers, i.e., KNN, ELM, SVM, and RELM, were compared with the enhanced classifiers, i.e., EKNN, EELM, ESVM, and ERELM, on the basis of classification accuracies. A single sensor approach was also compared with the belief function approach. Figure 8 shows the classification accuracy of fusion approaches using KNN. Figure 9 shows the result of EKNN and the comparison with other fusion approaches. It shows that the EKNN classifier outperformed the KNN classifier in terms of accuracy. NB and WMV did not perform well because they relied on finding the weights and probabilities of the classification results; whereas, to enhance the performance of the belief function fusion approach, EKNN was used. The classification and fusion accuracies of the three approaches using the KNN were lower than the EKNN.

In the original version of EKNN, it considers the distance information only in selecting the KNN. For the final class assignment decision, KNN does not play this role. On the basis of majority voting, class assignment is performed, and every vote of KNN has the same weights. This means that votes for the chosen KNN carry the same weights whether they are too close or too far from the test sample. If a value for the KNN chosen is even, then there is a chance of a tie in the voting. Because of this reason, KNN did not perform well. In the test sample, the evidence of each test sample regarding each KNN was combined to decide their classes. In EKNN, the distance between the samples was used to measure the weight of evidence. In the set of the KNNs, we have calculated the BBAs for each KNN. Then, we would have the BBAs corresponding to the k training samples. The class of the k nearest training samples increased our belief that the test sample belonged to that class. In EKNN, if a tie occurred, the class assignment decision could be handled by using the distance information. That is why the graph of EKNN showed high classification accuracies.

**Figure 10:**
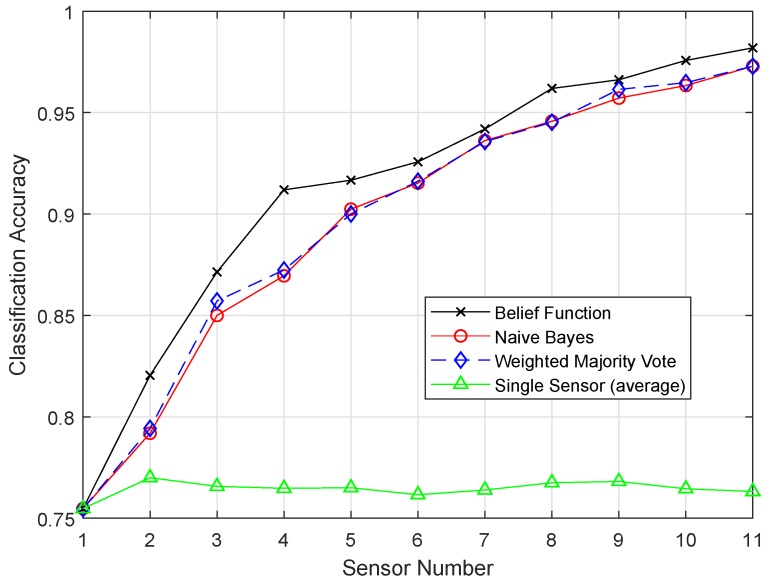
ELM.

**Figure 11:**
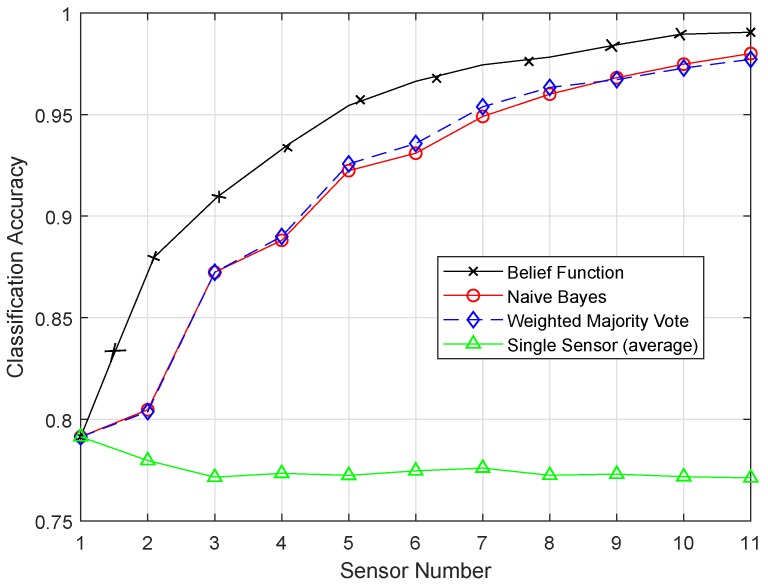
EELM.

Figure 10 and Figure 11 show the results of ELM and EELM, respectively. The belief function fusion approach with the EELM classifier beat the other fusion approaches, such as WMV, NB, and the existing belief function fusion approach. Using enhanced techniques, the classification and fusion accuracies were improved. In ELM, 20 hidden neurons were used, which resulted in the problem of over-fitting in classification. EELM started with a large amount of hidden nodes and checked the relevance of the class labels during the learning process. EELM ignored the irrelevant hidden nodes by checking the class labels in the learning process. That is why the EELM graph showed the highest classification accuracy, and the problem of over-fitting was avoided.

**Figure 12:**
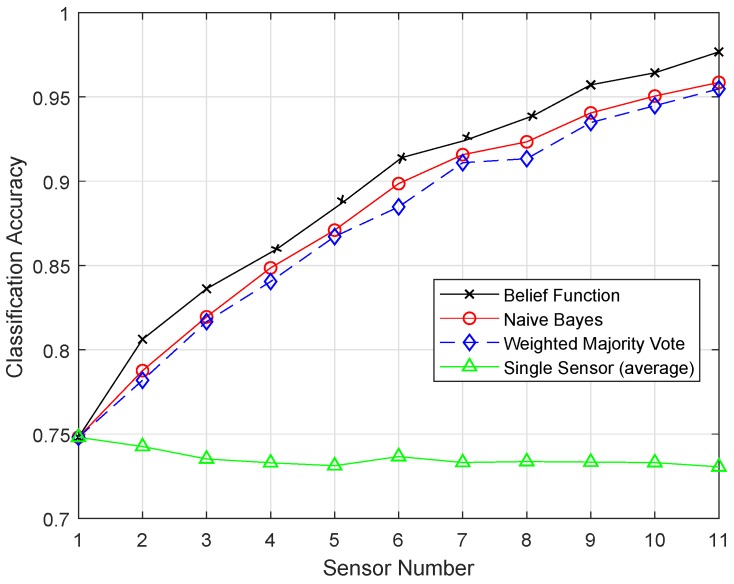
SVM.

**Figure 13:**
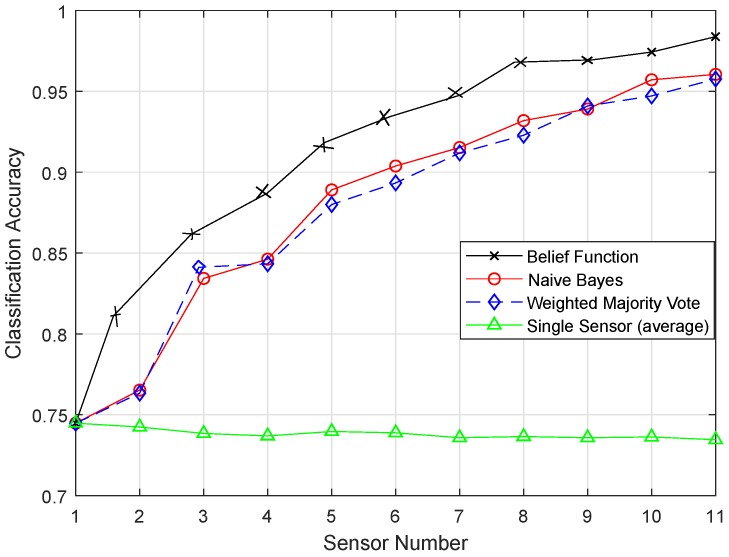
ESVM.

Figure 12 shows the result of the SVM classifier, and belief function outperformed the other two techniques. The Fitcsvm function was used for the SVM model. To improve the efficiency of the SVM classifier, efficient preprocessing was done to get good results. In Figure 13, the proposed ESVM gave better results than SVM and improved the performance of the belief function fusion approach. Choosing the best kernel function for SVM was the major key to its success. SVM provided the hyperplane in the feature space, and this hyperplane separated the data into two classes. SVM divided the data into two classes, however with the smallest intervals. Due to these smallest intervals, it showed less classification accuracy. Sigmoid function was used in SVM, which did not work well on the sensit vehicle dataset. ESVM used the radial basis kernel function, which classified the data accurately. A Sequential Minimal Optimization (SMO) solver was utilized to obtain the optimal decision boundary. First, 20, 40, and 60 samples of every class were used to train the SVM, separately. Every SVM model was tested using the last 40 samples of each class, which were not used for training. The radial basis kernel function and these parameters performed well on our dataset. Therefore, that is why ESVM performed well with increased classification accuracy.

**Figure 14:**
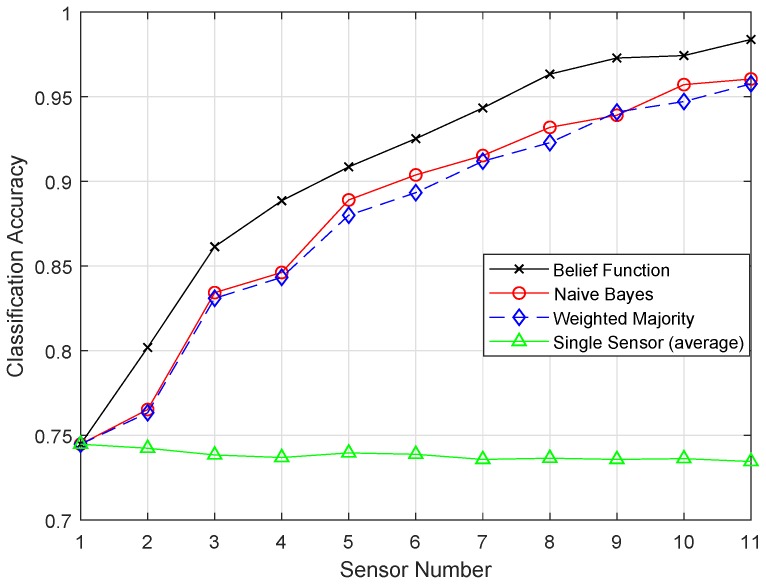
RELM.

**Figure 15:**
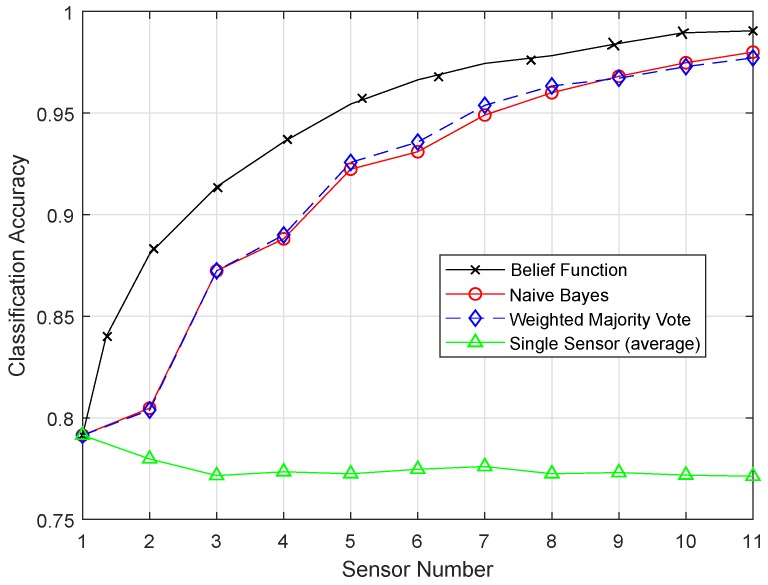
ERELM.

Figure 14 shows the result of the RELM method. Figure 15 shows that the belief function fusion approach with the ERELM classifier beat the other fusion approaches, WMV and NB, and the existing belief function fusion approach.

Features were extracted on the basis of the feedforward layer block. In training, the parameters of the extracted features were upgraded, and the results were adjusted according to the specified task. The backward recurrent layer and the training process followed were used to improve the classification process. RELM used the context neurons, and they were connected backward from the output to the input. Figure 15 shows the plot produced by ERELM, where the classification line shows the desired classification and fusion results. From all the simulation results, it can be concluded that the RELM technique outperformed the results of all the proposed enhanced techniques.

**Figure 16:**
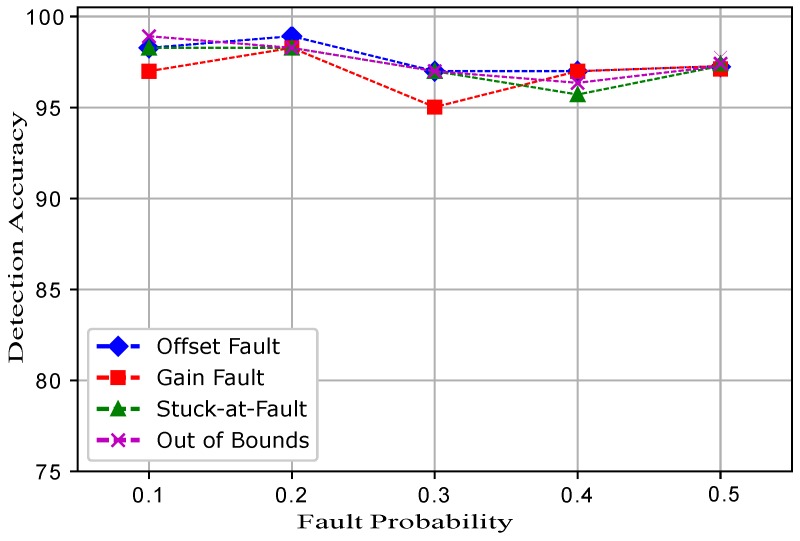
ERELM.

Figure 16 shows the detection accuracies of ERELM. The trend in Figure 16 shows that at only a 0.3 fault probability, the DA of RELM for the gain fault was 95%, and all the other faults showed the trend with a high accuracy of 97%. This indicates that between a 0.1 and a 0.2 fault probability, the DA was at 98%. The gain fault showed a different trend in the DA because in the equation of the gain fault, there was a constant multiplied with every reading. In the case of other faults, a constant was added, which had little impact on the detection accuracies of the classifiers. ERELM showed better results compared to EELM, ESVM, and EKNN and showed the highest DA. This is because the gains of RELM were transferred to the ERELM for training purposes in order to achieve a high accuracy. Weights in the output layer were calculated through the Moore–Penrose generalized inverse method.

(16) $$\hat{\beta }=H^{†}T$$

**Figure 17:**
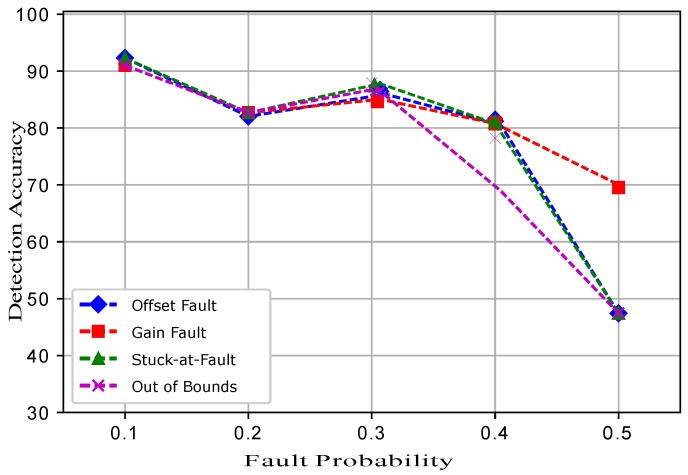
ESVM.

**Figure 18:**
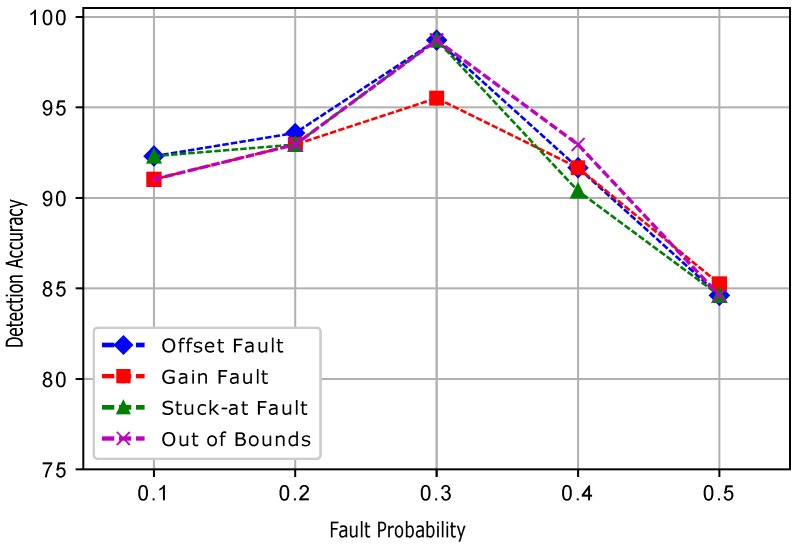
EELM.

**Figure 19:**
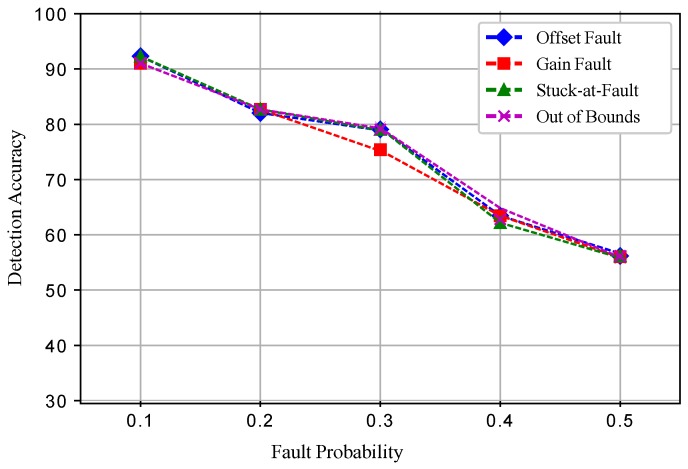
EKNN.

Figure 17 shows the detection accuracies of ESVM for all the four induced faults. At a 0.3 fault probability, the detection accuracies were increased and then showed a decreasing trend when the fault probability was increased. In ESVM, the average learning rates were obtained using the kernel functions. Because of the learning rate, changing features, and bordering data, ESVM gave lower performance than ERELM. Figure 18 shows that EELM also achieved better DA; however, when the fault probability increased, DA decreased gradually. Due to the lack of training samples and the decreased number of neurons, EELM’s performance was lower than ERELM and ESVM. Figure 19 shows the results of EKNN in terms of detecting accuracies for the induced faults. Because of the higher dimensionality of the data points and the Euclidean distance formula applied to our dataset, EKNN gave lower performance in comparison to the other enhanced classifiers; because the Euclidean distance formula gave equal weights to all features, and irrelevant features may interfere with the dissimilarity score. Detection accuracies for the ERELM ESVM, EELM, and EKNN classifiers are given in Table 3, Table 4, Table 5, Table 6 and Table 7, respectively.

Additionally, in WSNs, nodes have limited batteries to deal with heavy computations. In our proposed solution, the burden on the fusion center node and all other nodes involved in the network was reduced. Nodes will not send all the data to the fusion center, and only the BBAs are sent to the fusion center. All BBAs are combined through the combinational rule, and the fusion center will not have to perform complex computations. The combinational rule of the belief function fusion approach has performed the combination operation in the fusion center. In this way, the battery power of the fusion center node is saved. Fault detection and the proposed method have proven that network connectivity and data transmission will not be compromised in WSNs. An assumption was made that all the nodes had sufficient computational power to perform the classification and detection tasks [8].

## 7. Conclusions and Future Work

In this paper, we have improved the decision-based belief function fusion approach in WSNs. To improve the belief function fusion approach in WSNs, we enhanced four classifiers, namely EKNN, EELM, ESVM, and ERELM, for classification purposes. The proposed methods were compared with each other and with their original versions. Then, fault detection was performed for the belief function fusion approach in WSNs. Four types of faults were induced in the dataset: offset fault, gain fault, stuck-at fault, and out of bounds fault. The proposed classifiers were used to detect these faults, then their detection accuracies were analyzed and compared with each other. For comparison analysis of the proposed classifiers, the TPR was calculated. Another performance metric, ER, was also calculated for the proposed classifiers to analyze their detection accuracies. The results of all performance metrics showed that ERELM performed better compared to the other classifiers. Finally, the results were fused in the fusion center to analyze the overall performance of the belief function fusion approach in WSNs. For comparison analysis, the belief function fusion approach was compared with NB, WMV, and the existing belief function fusion approach. Experimental results showed that our improved belief function fusion approach beat NB, WMV, and the existing belief function fusion approach. In the future, we will find better ways to calculate decision reliability to improve fusion results. We will apply the proposed solution in other multi-class fusion applications, like remote sensing, image fusion, and multi-symbol signal modulation.

## Figures and Tables

**Table 1:** Related work.

Classifiers	Goals	Limitations	Techniques and Methods
KNN, NB, neural network, decision tree [3]	Classification of forest high-resolution remote-sensing image	No association between data mining and conflict management	Generic framework and automatic method for weighting factors
Data distribution-based intuitionistic fuzzy set construction algorithm [4]	Multi-attribute decision fusion model	Negative and positive non-ideal solutions are not elaborated	Intuitionistic fuzzy set
Multi-input and multi-output decision fusion classifier [5]	Needed amount of energy for WSN	Performance decreases with high SNR	On-off keying scenario
Fourier-based, stream-based classifiers [6]	Handle energy dissipation	Did not exploit parallelism	Deep tree-based structures
CNN, SVM [9]	Golf swing classification method	No discussion about relevancy, redundancy for sensors	Architecture of vanilla convolutional neural network
Gaussian NB [10]	Processing of sensor dataset using various machine learning algorithms	Feature selection is not used	Supervised machine learning
PCA, ANN, SVM [11]	Classification of ECG signal	No feature selection	Adaptive filter
ELM [12]	Enhanced ELM	Applications of the proposed methods are not discussed	Regularized-ELM, $L_{2}$-norm-optimized ELM and kernel-ELM
MLP [13]	To detect state of charge	Their is no regression method used	Structured MLP architecture
KNN [14]	To predict data accurately	Temporal correlation is lacking	Linear regression model to describe the spatial correlation
KNN [15]	To develop a reliable spectrum sensing scheme	No feature selection or extraction	Majority voting
SVM [16]	Fault detection	Regression is not elaborated	Decision tree classification protocol
SVM [17]	Fault detection	Overfitting problem	K-fold cross-validation technique
SVM, NB, and gradient lifting decision tree [18]	Fault detection	No DA	Non-linear mapping algorithm

**Table 2:** ER.

Fault Types	EKNN	EELM	ESVM	ERELM
Offset Fault	4.52%	4.79%	4.81%	4.86%
Gain Fault	4.90%	5.23%	5.96%	6.13%
Stuck-at Fault	4.77%	5.13%	6.9%	5.65%
Out of Bounds	4.81%	4.91%	5.82%	6.77%

**Table 3:** DA with 10% induced faults.

Fault Types	ERELM	ESVM	EELM	EKNN
Offset Fault	97.9%	93.3%	92.3%	92.2%
Gain Fault	96.5%	90.0%	91.5%	80.0%
Stuck-at Fault	97.3%	91.6%	95.7%	90.5%
Out of Bounds	98.8%	98.4%	91.6%	90.4%

**Table 4:** DA with 20% induced faults.

Fault Types	ERELM	ESVM	EELM	EKNN
Offset Fault	98.8%	94.1%	81.2%	82.0%
Gain Fault	98.1%	93.5%	84.5%	81.2%
Stuck-at Fault	98.2%	93.0%	83.5%	81.0%
Out of Bounds	98.0%	93.2%	83.6%	81.4%

**Table 5:** DA with 30% induced faults.

Fault Types	ERELM	ESVM	EELM	EKNN
Offset Fault	97.4%	97.9%	83.0%	80.0%
Gain Fault	95.0%	95.0%	82.8%	73.0%
Stuck-at Fault	97.1%	98.9%	83.3%	80.2%
Out of Bounds	97.5%	97.9%	82.9%	80.1%

**Table 6:** DA with 40% induced faults.

Fault Types	ERELM	ESVM	EELM	EKNN
Offset Fault	97.2%	93.2%	81.1%	62.8%
Gain Fault	97.1%	93.2%	81.0%	62.4%
Stuck-at Fault	95.8%	90.5%	80.4%	61.0%
Out of Bounds	97.0%	94.8%	70.0%	63.2%

**Table 7:** DA with 50% induced faults.

Fault Types	ERELM	ESVM	EELM	EKNN
Offset Fault	97.8%	84.8%	48.3%	58.8%
Gain Fault	97.0%	85.2%	70.1%	58.8%
Stuck-at Fault	97.3%	84.5%	48.3%	58.9%
Out of Bounds	97.4%	84.4%	48.9%	58.9%

## Abbreviations

The following abbreviations are used in this manuscript:

BBA	Basic Belief Assignment
CNN	Convolutional Neural Network
ELM	Extreme Learning Machine
EKNN	Enhanced K-Nearest-Neighbor
EELM	Enhanced Extreme Learning Machine
ERELM	Enhanced Recurrent Extreme Learning Machine
ESVM	Enhanced Support Vector Machine
EKNN	K-Nearest-Neighbor
MLP	Multilayer Perceptron
NB	Naive Bayes
RELM	Recurrent Extreme Learning Machine
SVM	Support Vector Machine
TPR	True Positive Rate
WMV	Weighted Majority Vote
WSN	Wireless Sensor Network
